# Supplementary material for: Nanomanufacturing of silicon surface with a single atomic layer precision via mechanochemical reactions
Source: Nat Commun. 2018 Apr 18;9:1542. doi: 10.1038/s41467-018-03930-5 (PMC5906689; doi:10.1038/s41467-018-03930-5)
Supplement: Supplementary file 1 — Supplementary Information [file 41467_2018_3930_MOESM1_ESM.docx]

**Supplementary Note 1. Characterization of SiO_2_ tip used for manufacturing**

The SiO_2_ microspherical tip used for mechanochemical nanomanufacturing was characterized by scanning electron microscopy (SEM). Supplementary Figure 1 shows the SEM images of the SPM cantilever (Supplementary Figure 1a) and silica microsphere (Supplementary Figure 1b). The radius of silica micro-ball was measured to be 1.25 μm.


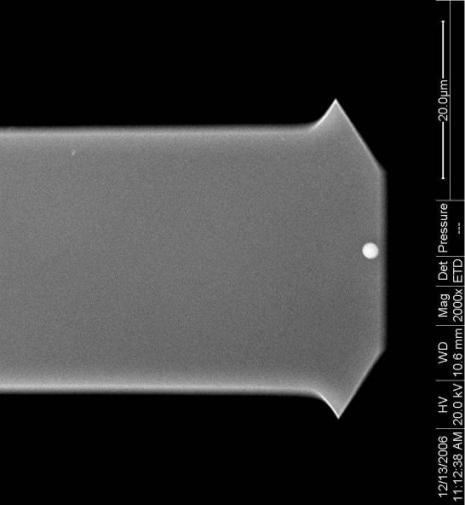


10 μm


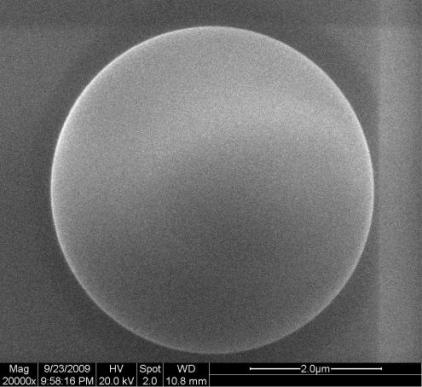


500 nm

(a)

(b)

**Supplementary Figure 1.** SEM image of SiO_2_ microsoherical tip

**Supplementary Note 2. Calibration of SPM by scanning single graphite layer**

The topographic imaging precision of SPM in the vertical direction was calibrated by scanning a single layer step edge of graphite. As shown in Supplementary Figure 2, the height of single graphite layer was measured as 3.4 ± 0.2 Å. The theoretical value of a single layer is 3.4 Å, so the error in height of SPM topographic images is less than 6%.

(b)

(a)


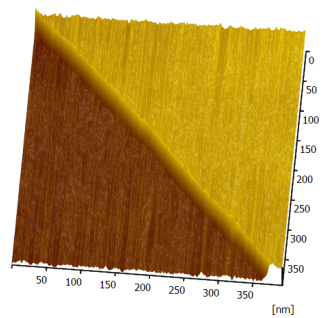


3.4 ± 0.2 Å

**Supplementary Figure 2.** SPM image (a) and cross-section profile (b) of single layer graphite. The thickness of monolayer was measured as 3.4 ± 0.2 Å by a sharp Si_3_N_4_ tip with a radius less than 20 nm.

**Supplementary Note 3. Load dependent manufacturing**

After mechanochemical etching tests at various applied loads in humid air (RH = 75% ± 2%), it was found that the lowest contact load at which removal of Si atoms was 0.3 µN, as shown in Supplementary Figure 3a. Based on the DMT contact mechanics, this load corresponds to a contact stress of 247 MPa. Below this value, no obvious material removal was detected on the Si surface (0.15 µN in Supplementary Figure 3a). Thus, 247 MPa could be taken as an upper limit of the critical stress (*σ*_0_).

The increase of contact stress above *σ*_0_ facilitated the mechanochemical removal of Si atoms (Supplementary Figure 3a). Supplementary Figure 3b shows the mechanochemical-etch rate as a function of average DMT contact stress. The contact stress (*σ*) dependence of volume loss rate (*δ*) followed the Arrhenius-type relationship (inset in Supplementary Figure 3b), which implied that the overall process can be modeled as a stress-assisted thermal activation process^1^. If the effect of shear stress is viewed as lowering the thermal activation barrier, then the Arrhenius equation can be expressed as:

$\delta=bf_{0}\exp(-\frac{\Delta U_{\text{act}}}{k_{\text{B}}T})\exp(\frac{\sigma\Delta V_{\text{act}}}{k_{\text{B}}T})=C\exp(\frac{\sigma\Delta V_{\text{act}}}{k_{\text{B}}T})$ (1)

Here, *f*_0_ is an effective attempt frequency, *b* is a lattice parameter, Δ*U*_act_ is a thermal activation barrier, Δ*V*_act_ is a critical activation volume, *k*_B_ and *T* are Boltzmann’s constant and the absolute temperature, respectively. Since *f*_0_ is constant at a given sliding speed, *b* does not vary unless the substrate is changed, and Δ*U*_act_ could be assumed to be constant for a given reaction, the *bf*_0_exp(-Δ*U*_act_*k*_B_^-1^*T*^-1^) term can be lumped into a constant term *C* in equation (1). Fitting the data in Supplementary Figure 3b with equation (1) gave the activation volume Δ*V*_act_ of ~32.7 Å^3^. This is the activation volume with respect to the normal stress; if only the tangential shear stress is considered, this value should be divided by the friction coefficient.

**Supplementary Figure 3.** Load dependence of atomic removal on Si surface. (a) Worn topographies and the corresponding cross-section profiles of Si against a silica sphere at load ranging from 0.15 µN to 2.5 µN. The sliding speed (*v*) was 4 µm s^-1^, and RH was 75 ± 2%. (b) Contribution of contact stress *σ* to the volume loss rate of the Si substrate. *σ*_0_ is the critical contact stress below which material removal could not be detected. Inset shows that the contact stress (*σ*) dependence of volume loss rate follows an exponential relationship. The error bars are estimated from four repeated experiments.

**Supplementary Note 4. Atomic layer removal in TEM observation**

The high resolution atomic structure of the mechanochemically-etched area was analyzed with TEM (Tecnai G2, FEI, USA). The cross-section of the scanned region was prepared with focused ion beam (FIB) technique (Nanolab Helios 400S, FEI, Holland). Before the FIB milling, the Si surface was deposited with a passivation layer of epoxy polymer. Note that platinum layer, which is typically used for this purpose, can induce decrystallization of the substrate material due to the impact of high energy particles during physical vapor deposition. After scanning with the SiO_2_ microsphere for 50 reciprocating sliding cycles at *F*_n_ of 2300 nN, *v* of 16 µm s^-1^ and RH of 75 ± 2% in a line-scratch mode, a ~16 Å deep trench was produced on the silicon surface (Supplementary Figure 4), which corresponded to twelve atomic layers (Fig. 2 in the main text).

(a)

(b)

**Supplementary Figure 4.** SPM image (a) and cross-section profile (b) of the wear scar on the silicon surface used as the sample of TEM observation. The wear scar with the maximum depth of ~1.63 nm was formed by using line-scratch method after 50 reciprocating robbing cycles at *F*_n_ of 2.3 µN (*σ* equals to 571 MPa), *v* of 16 µm s^-1^ and RH of 75 ± 2%.

The cross-section of a deeper etch line was analyzed with TEM. After 200 reciprocating cycles of sliding of the SiO_2_ tip at a load of 3 µN, a groove with ~4.6 nm in depth and ~74.7 nm in width was formed on the Si surface. The good agreement between SPM and TEM images of wear depth and cross-section profile is shown in Supplementary Figures. 5a and b. High-resolution TEM images shown in Supplementary Figures 5c and d demonstrate that, similar to Fig. 2 reported in the main text, both ends of the wear scar exhibit the layered structure of the silicon lattice. When the load was decreased to 0.3 µN (line-scratch model), the wear scar with a depth of 2.8 Å was generated after 10 sliding cycles in humid air (75 ± 2% RH). TEM images show removal of double atomic layers from the Si surface (Supplementary Figure 6c). Scars I and III shown in Supplementary Figures 6a and b were made as reference marks to find the location of scar II with TEM. Supplementary Figures 6d and e show the formation of atomic layer structure at both edges of the wear scar.

**Supplementary Figure 5.** Layered structure observed in the cross-section of wear scar with 4.63 nm in depth and 74.4 nm in width. (a) SPM image and the corresponding cross-section profiles of the wear scar formed after 200 reciprocating sliding cycles at *F*_n_ of 3 µN, *v* of 10 µm s^-1^ and RH of 75 ± 2%. (b) TEM observations of the cross-section of wear scar. (c) and (d) respectively show the high-solution TEM images of Si crystalline structure on the left and right ends (inside white dashed frame in b) of the wear scar.

**Supplementary Figure 6.** High-solution TEM images of cross-section of the scanned area showing the formation of layered structures on the Si surface. (a) SPM image of the wear scars: scar I (depth is around 60 Å) is a mark for locating in sample preparing; scar II is the high-solution TEM imaged wear scar; and scar III (depth is around 5.5 Å) is a mark for locating in TEM imaging. (b) TEM image of the scars II and III. (c) TEM image of scar II. (d) and (e) show the high-solution TEM images of the edge regions of scar II.

**Supplementary Note 5. Wear of silicon against SiO_2_ tip in vacuum (less than 10^-6^ Torr)**

When the mechanochemical etching was attempted with the SiO_2_ tip at an average contact pressure of 247 MPa in vacuum (less than 10^-6^ Torr), no wear was observed on the Si surface after scanning. When the average contact pressure was increased to 630 MPa (*F*_n_ was 3 μN), a hillock (protrusion) with a height of 0.3 ± 0.1 nm was formed on the silicon surface, as shown in Supplementary Figure 7. This behavior is consistent with the results in previous studies^2,3^.

(b)

(a)


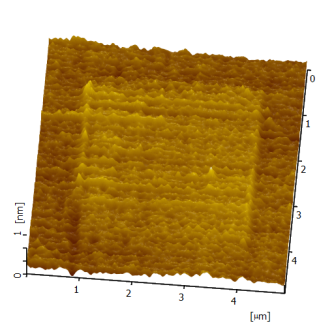


**Supplementary Figure 7.** SPM image (a) and cross-section profile (b) of wear area on silicon surface after slid against SiO_2_ tip in vacuum. The scanning area was 3 × 3 µm^2^. The sliding cycles were 10 and the sliding speed was 1.2 µm s^-1^. The normal load was 3 µN.

**Supplementary Note 6. Mechanical removal on silicon surface against diamond tip in humid air**

When the counter-surface (scanning tip) was changed to a diamond tip, the formation of hillock, instead of removal of materials, was observed even in humid air (RH was 70 ± 2%). As shown in Supplementary Figure 8, a protruded hillock with a height of about 1 nm was formed after scanning 5 times at a contact pressure of 9.5 GPa. Supplementary Figure 9 shows a typical TEM image of the hillock formed on the Si surface after the line scanning test. It can be seen that the hillock is composed of an amorphous structure of several nanometers in thickness, which is caused by the mechanical interaction between the tip and the Si surface. When the contact pressure was increased to 13 GPa, the wear of Si occurred, as shown in Supplementary Figure 10. Supplementary Figure 11a shows the TEM image of a groove formed on the Si surface under the line scanning test. The groove with a depth of 8 nm (inset SPM image in Supplementary Figure 11) was formed when the diamond tip (radius was 2 μm) was used for scanning at 13 GPa load. The selected-area diffraction (SAD) patterns indicated that the top 200 nm region is amorphous (Supplementary Figure 11b). Beneath the amorphous layer, a crystalline zone with slip lines and stacking faults was observed (Supplementary Figure 11c).

(b)

)

(a)


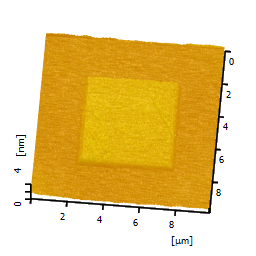


**Supplementary Figure 8.** SPM image (a) and cross-section profile (b) of wear area on silicon surfaces after slid against a diamond tip under the contact pressure of 9.5 GPa. The tip radius was 250 nm. The scanning area was 5 × 5 µm^2^. The sliding cycles were 5 and the sliding speed was 10 µm s^-1^.

**Supplementary Figure 9.** XTEM image showing the cross-sectional structure of hillock on the silicon surface. Inset picture shows the corresponding SPM image of hillock formed against diamond tip with the line sliding model. The contact pressure was 9.5 GPa. The sliding cycles were 100 and the sliding speed was 10 µm s^-1^.

(b)

)

(a)


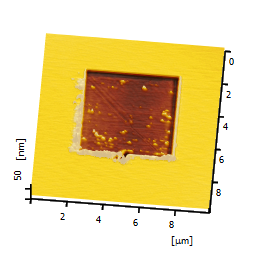


**Supplementary Figure 10.** SPM image (a) and cross-section profile (b) of wear area on the silicon surface after slid against a diamond tip under the contact pressure of 13 GPa. The tip radius was 250 nm. The scanning area was 5 × 5 µm^2^. The sliding cycles were 5 and the sliding speed was 10 µm s^-1^.


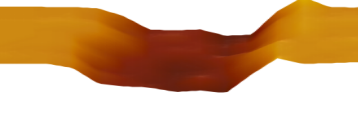

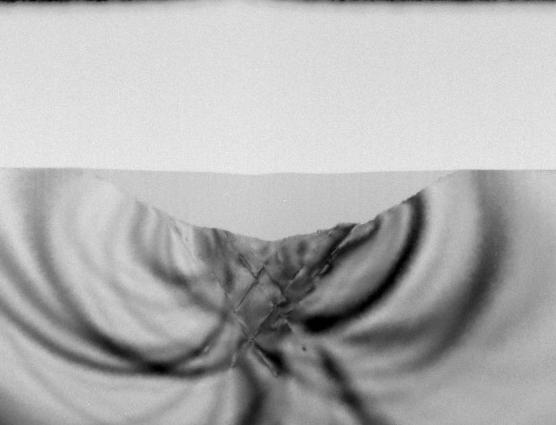

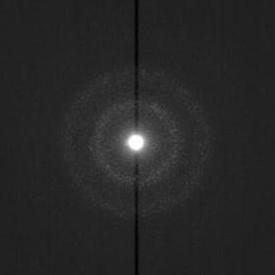


(b)

(a)

Passivation layer

8 nm

0.2 µm

(b)


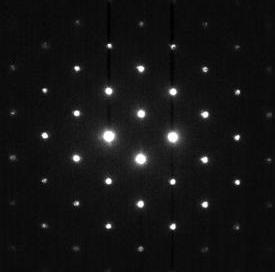


(c)

(c)

0.2 µm

Si

**Supplementary Figure 11.** (a) XTEM image showing the cross-sectional structure of the groove on silicon surface. Inset shows the corresponding SPM image of groove formed against diamond tip (radius is 2 μm) under the contact pressure of 13 GPa. The sliding cycle was 1 and sliding speed was 10 µm s^-1^. (b) and (c) respectively show the SAD patterns from the amorphous area and Si substrate with defect.

**Supplementary Note 7. MD simulations**

In MD simulations, the Si(100) substrate with dimensions of 69.12 × 69.12 × 30.09 Å (24 Si atomic layers with 7776 atoms) was divided into three layers, including bottom-most fixed layer, thermostat layer in the middle and free layer at the top. The hemispherical SiO_2_ tip (radius was 30 Å), with inverse order of the three layers, was cleaved from the initial amorphous silica structure produced from a melt quench process of a bulk quartz silica crystal, and it was fully terminated with hydroxyls. A liquid layer with 1600 water molecules (around 1.0 nm thickness) was constructed to cover the Si substrate (Supplementary Figure 12).


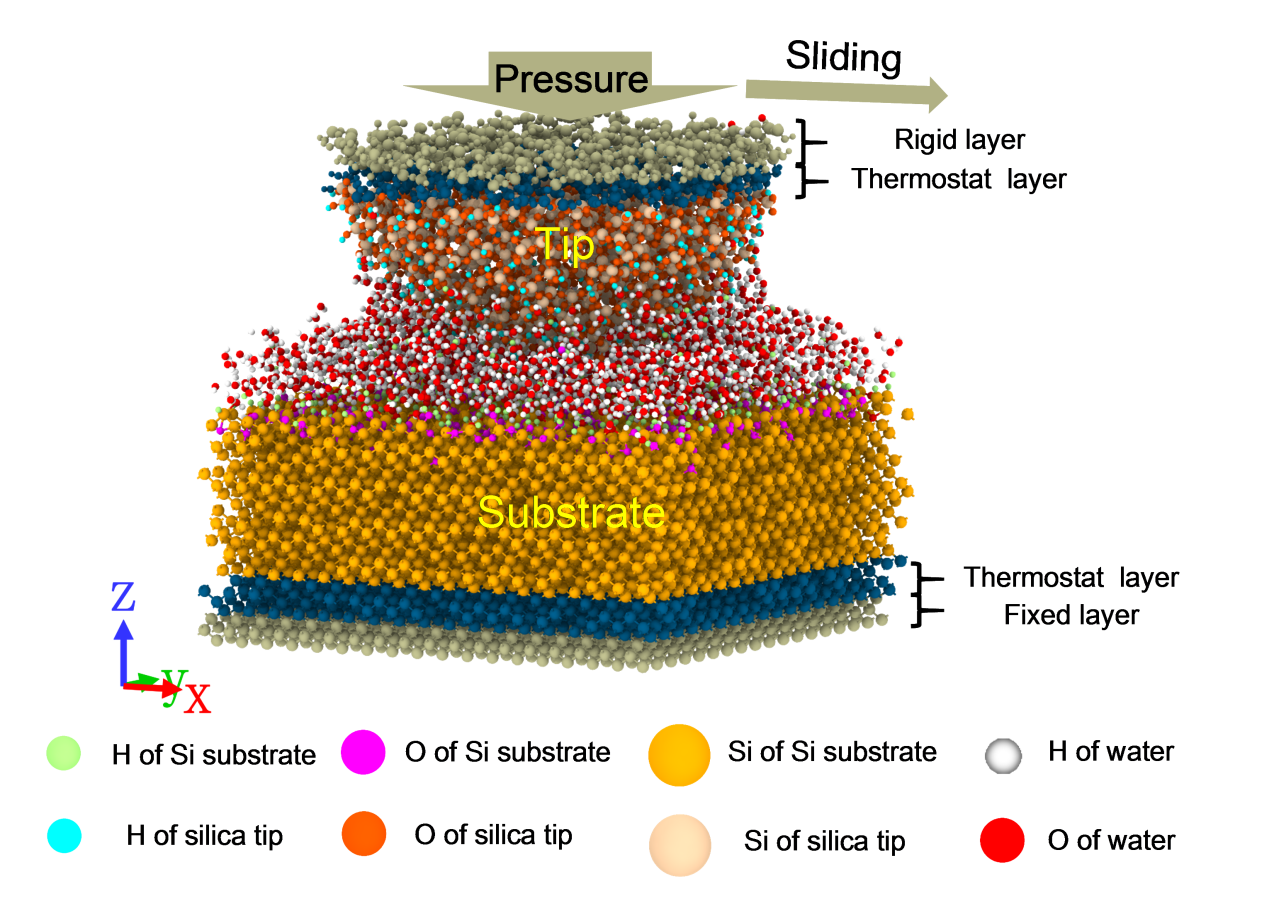


**Supplementary Figure 12.** Sliding model of the silicon substrate and silica tip under water condition.

**Supplementary Note 8. Scanning Auger Electron Spectroscopy (sAES) of the mchanochemically-etched surface**

The degree of surface oxidation of the mechanochemically-etched area was analyzed with a scanning Auger nanoprobe with micro-area analysis function (ULVAC, PH1710, JAPAN). In order to find the nanofabricated area using the secondary electron imaging mode of the sAES system, two mechanical scratch lines (around 90 nm deep) were first marked on the Si wafer using a diamond tip; then, a 7 µm × 7 µm area between two reference lines was contact scanned with a SiO_2_ microsphere tip to induce mechanochemical etching (Supplementary Figure 13a). The contact load was 2.5 μN, the scan speed was 14 μm s^-1^, and the scan line density was ~73 lines µm^-1^. After 6 reciprocating cycles of the contact scan over the 7 µm × 7 µm region, the mechanochemical etch depth was found to be about1.8 nm (Supplementary Figure 13b).

Supplementary Figure 13c compares the O KLL and Si KLL Auger spectra measured for the mechanochemically-etched area and the surrounding area of the Si(100) wafer surface. Also shown are the Auger spectra of the freshly HF-etched surface and the as-received wafer surface. The presence of plasmon peaks (1605 eV and 1587 eV) of the Si KLL peak (1622 eV)^4^ indicates that the oxide thickness is very small (even for the native oxide of the as-received sample). The O KLL peak positions of the HF-etched surface is slightly higher than that of the as-received sample; it is known that the O KLL peak position is lower for the oxide than the chemisorbed oxygen.^5,6^ Similarly, it was reported that the Si KLL peak position of silica is slightly lower than that of the elemental silicon.^4^ These data support that the native oxide is completely removed through the HF-etching process before the mechanochemical nano-fabrication test.

Supplementary Figure 13d compares the O KLL (513 eV) / Si KLL (1622 eV) intensity ratios of the four different surfaces; since the surface is not homogeneous along the AES probe depth, the conversion of the intensity ratio to relative concentration was not attempted. The as-received sample shows a relatively high O/Si ratio (0.68) due to the presence of native oxide layer. Upon removal of the native oxide layer with HF, the O/Si ratio decreases to 0.17. A small amount of surface oxidation was inevitable since the sample was handled in ambient air. The surrounding region of the mechanochemically-etched region has a bit higher O/Si ratio (0.22), since the surface was exposed to the ambient air for a longer period of time during the SPM experiment. It is important to note that the mechanochemically-etched region shows the O/Si ratio of 0.38, which is larger than the surrounding region (O/Si ≈ 0.22) that was not mechanically contacted, but exposed to the ambient condition for the same amount of time. The higher O/Si ratio of the mechanochemically-etched region is the consequence of surface reactions between the topmost silicon atoms and the water molecules adsorbed from the ambient air induced by interfacial shear with the SiO_2_ counter-surface. Without the shear with SiO_2_, thermal oxidation of the hydrogen-terminated Si surface (prepared by HF etching) at the room temperature occurs at a slower rate.

**Supplementary Figure 13.** (a) Secondary electron image of the mechanochemically-etched area (7 µm × 7 µm) and two mechanically-scribed reference lines. (b) SPM topographic image of the mechanochemically-etched region. (c) AES spectra of the mechanically-etched region, the surrounding area, the freshly HF-etched surface, and the as-received surface with native oxide layers. (d) Intensity ratio of O KLL (513 eV) and Si KLL (1622 eV) peaks.

**Supplementary References**

Jacobs, T. D. B. & Carpick, R. W. Nanoscale wear as a stress-assisted chemical reaction. *Nat. Nanotechnol.* **8**, 108-112 (2013).

Yu, J. X. *et al.* Role of tribochemistry in nanowear of single-crystalline silicon. *ACS Appl. Mater. Interfaces* **4**, 1585-1593 (2012).

Yu, B. J. *et al.* Friction-induced nanofabrication on monocrystalline silicon. *Nanotechnology* **20**, 465303 (2009).

Thomas. S. Electron-irradiation effect in the Auger analysis of SiO_2_. *J. Appl. Phys.* **45**, 161 (1974).

Wagn er, C. D., Zatko, D. A., Raymond, R. H. Use of the oxygen KLL Auger lines in identification of surface chemical states by electron spectroscopy for chemical analysis. *Anal. Chem.* **52**, 1445-1451 (1980).

Berjoan, R., Rodriguez, J. Sibieude, F. AES study of the SiO_2_/SiC interface in the oxidation of CVD β-SiC. *Surface Science* **271**, 237-243 (1992).
