# Supplementary material for: Nanomanufacturing of silicon surface with a single atomic layer precision via mechanochemical reactions
Source: Nat Commun. 2018 Apr 18;9:1542. doi: 10.1038/s41467-018-03930-5 (PMC5906689; doi:10.1038/s41467-018-03930-5)
Supplement: Supplementary file 3 — Description of Additional Supplementary Files [file 41467_2018_3930_MOESM3_ESM.docx]

**Description of Additional Supplementary Files**

File Name: Supplementary Movie 1

Description: Full recovery of the crystalline lattice structure without subsurface damage after load is released.
